# The Rhododendron Chrysanthum Pall.s’ Acetylation Modification of Rubisco Enzymes Controls Carbon Cycling to Withstand UV−B Stress

**DOI:** 10.3390/biom14060732

**Published:** 2024-06-20

**Authors:** Meiqi Liu, Fushuai Gong, Wang Yu, Kun Cao, Hongwei Xu, Xiaofu Zhou

**Affiliations:** Jilin Provincial Key Laboratory of Plant Resource Science and Green Production, Jilin Normal University, Siping 136000, China

**Keywords:** lysine acetylome, chlorophyll fluorescence, photosynthesis, carbon fixation, *R. chrysanthum*

## Abstract

Lysine acetylation of proteins plays a critical regulatory function in plants. A few advances have been made in the study of plant acetylproteome. However, until now, there have been few data on *Rhododendron chrysanthum* Pall. (*R. chrysanthum*). We analyzed the molecular mechanisms of photosynthesis and stress resistance in *R. chrysanthum* under UV−B stress. We measured chlorophyll fluorescence parameters of *R. chrysanthum* under UV−B stress and performed a multi−omics analysis. Based on the determination of chlorophyll fluorescence parameters, *R. chrysanthum* Y(NO) (Quantum yield of non−photochemical quenching) increased under UV−B stress, indicating that the plant was damaged and photosynthesis decreased. In the analysis of acetylated proteomics data, acetylated proteins were found to be involved in a variety of biological processes. Notably, acetylated proteins were significantly enriched in the pathways of photosynthesis and carbon fixation, suggesting that lysine acetylation modifications have an important role in these activities. Our findings suggest that *R. chrysanthum* has decreased photosynthesis and impaired photosystems under UV−B stress, but NPQ shows that plants are resistant to UV−B. Acetylation proteomics revealed that up- or down-regulation of acetylation modification levels alters protein expression. Acetylation modification of key enzymes of the Calvin cycle (Rubisco, GAPDH) regulates protein expression, making Rubisco and GAPDH proteins expressed as significantly different proteins, which in turn affects the carbon fixation capacity of *R. chrysanthum*. Thus, Rubisco and GAPDH are significantly differentially expressed after acetylation modification, which affects the carbon fixation capacity and thus makes the plant resistant to UV−B stress. Lysine acetylation modification affects biological processes by regulating the expression of key enzymes in photosynthesis and carbon fixation, making plants resistant to UV−B stress.

## 1. Introduction

*Rhododendron chrysanthum* Pall. (*R. chrysanthum*) is a rare resource for the study of abiotic stress in plants since it grows permanently in the alpine zone of Changbai Mountain, which has strong radiation and high altitude [1]. *R. chrysanthum* has demonstrated adaptation to UV−B radiation and other abiotic stresses over the course of its lengthy growth and evolution [2].

Among the ultraviolet rays in sunlight that cause the most concern is medium−wave ultraviolet radiation (UV−B, 280–320 nm) [3]. Because of their sessile growth habit, plants are inevitably exposed to UV−B stress. Most plants that are exposed to UV−B radiation will react by producing stress [3]. According to pertinent research, UV−B stress can harm plants in a number of ways, including slowing down their growth, inhibiting photosynthesis, oxidative damage, and rupturing the integrity of vital macromolecules [4,5,6]. Green plants’ rate of photosynthetic activity may be significantly lowered by UV−B stress [7], and parameters connected to chlorophyll fluorescence are crucial markers of photosynthetic characteristics [8]. The kale (*Brassica oleracea* L.) showed a decrease in Fv/Fm (maximum photochemical efficiency of PSII) and fluorescence transient parameters (RC/CS, ABS/CS, TR_O_/CS) with UV−B stress [9]. The maximum photochemical efficiency of PSII, or the ratio of chlorophyll fluorescence parameter Fv/Fm, dramatically dropped in *Olea europaea* (L.) under long-term UV−B stress [10]. Due to its special growing environment, *R. chrysanthum* was used as the experimental material in this study. In order to ascertain the effects of UV−B radiation on photosynthesis and the ideal UV−B radiation dosage for *R. chrysanthum*, we carried out a pertinent study. We also determined the duration of UV−B radiation, which was 48 h, and found that *R. chrysanthum* photosynthesis was inhibited by UV−B but not by UV−A [2]. After UV−B treatment, we also conducted the measurement of chlorophyll fluorescence parameters, and the Fv/Fm and Fv/Fo decreased dramatically, revealing that photo-synthesis was impeded and the photosystem was injured [11].

Proteins’ post-translational modifications (PTMs) can alter their activities, subcellular locations, and interaction partners, among other aspects of protein functional stability regulation [12]. One of the most common PTMs in both prokaryotes and eukaryotes is lysine acetylation (Kac) modification [13,14]. After undergoing extensive research, it was discovered that kac was involved in numerous significant processes, including the germination of seeds, the development of organs, blooming, stress response, and leaf senescence [15,16,17,18,19,20]. During the dormancy release process in poplar, acetylation modification of enzymes in the primary metabolic pathway is an important strategy for breaking the dormancy of bloom buds [21]. The acetylation of essential enzymes for starch biosynthesis to adapt to drought stress is revealed by acetylation proteomics analysis of growing wheat kernels under conditions of water deficit [22]. The OsHYPK-NatA complex plays a crucial role in coordinating rice development and stress responses, primarily through its dynamic regulation of NatA-mediated N-terminal acetylation and global protein turnaround [23]. *Sorghum bicolor* HDAC SbHDT701 enhances acetylation modifications to regulate stress response [24]. LysAc may have a major and previously unknown regulatory role in many nonhistone proteins that are involved in pathways and functions in Arabidopsis and other plants [25]. Some proteins, especially those involved in photosynthesis, glycolysis, and secondary metabolism, may be lysine-acetylated to control particular metabolic processes in tea leaves [26]. Our earlier study revealed that *R. chrysanthum’s* ability to photosynthesise was hampered by UV−B radiation, but that UV−B damage to plants could be mitigated by acetylating the PSII protein. This study did not fully explore the photosynthetic carbon cycle process, which is crucial to understanding biological processes [11].

It has been investigated and determined how the *R. chrysanthum* photosystem II protein reacts to UV−B stress. But the examination initially concentrated on physiology, biochemistry, and photosystem aspects [11]. However, plants’ ability to sequester carbon is also very significant. Thus, we conducted an acetylation-modified proteomics analysis of *R. chrysanthum* in order to gain a better understanding of the molecular mechanism of UV−B tolerance. This analysis offers important insights for future research on the molecular mechanism of UV−B radiation resistance.

## 2. Materials and Methods

### 2.1. Plant Material and Treatment

*R. chrysanthum was* kept in a climate chamber designed to mimic the conditions of a high mountain environment [2], and in the artificial climate chamber following storage, *R. chrysanthum* was continuously grown and cultured under white fluorescent lights. In the climate chamber, *R. chrysanthum* was cultivated under 50 µmol (photon) m^−2^ s^−1^ white fluorescent lights. And the following conditions were present in the artificial climate chamber: 18 °C for 14 h of light and 16 °C for 10 h of darkness. UV−B and photosynthetically active radiation (PAR) were used in this study. Two groups of *R. chrysanthum* were created: the CG group received a 48-h treatment with PAR, while the BG group received a 48-h treatment with PAR+UV−B. Each group received three biological duplicates (three repetitions for the CG group and three repetitions for the BG group) to guarantee adequate coverage. PAR radiation was performed by placing a 400 nm filter (Edward, Filter Long 2IN SQ, Barrington, NJ, USA) on the culture flasks (effective irradiance of 50 μmol m^−2^ s^−1^), and PAR+UV−B radiation was performed by placing a 295 nm filter (Edward, Filter Long 2IN SQ, Barrington, NJ, USA) (effective irradiance of 2.3 W m^−2^).

### 2.2. Measurement of Chlorophyll Fluorescence

*R. chrysanthum* leaves were used to evaluate the induction features of chlorophyll fluorescence using the Imaging-PAM Maxi (HeinzWalz, Effeltrich, Germany). The plants were treated for 20 min in the dark prior to measuring them. We measured the fluorescence parameters. Actual photochemical quantum yield of PSII Y(II); Maximum quantum yield of PSII (Fv′/Fm′); Electron transport rate (ETR), non-photochemical quenching coefficient (NPQ), photochemical quenching coefficient (qL), and quantum yield of uncontrolled energy dissipation of photosystem II Y(NO).

### 2.3. Quantitative Proteomic Study of Acetylated Modification

#### 2.3.1. Protein Extraction

This experiment’s acetylated proteomics were provided by Jingjie PTM Hangzhou Co Inc. BioLab (Hangzhou, China). Using a high−intensity ultrasonic processor (Scientz, Ningbo, China), *R. chrysanthum* was ground and pulverized in liquid nitrogen, transferred to centrifuge tubes, and sonicated three times on ice in a lysis buffer (8 M urea, 2 mM EDTA, 10 mM DTT, and 1% protease inhibitor cocktail). After centrifugation at 5500× *g* for 10 min at 4 °C, the supernatant was centrifuged for 3 min at 4 °C. Proteins in the supernatant were centrifuged for 3 min at 4 °C, and it was then precipitated with 15% TCA for 4 h at −20 °C. The rest of the sediment was washed three times with acetone after chilling in a refrigerator. Finally, the proteins were redissolved in buffer, the buffer consisted of 8 M urea, 100 mM TEAB, pH 8.0. And in order to measure the protein concentration in the supernatant, it was estimated using the 2-D Quantay Analysis Kit (Cytiva 80-6483-56).

#### 2.3.2. Trypsin Digestion

TCA at a concentration of 20% (*m*/*v*) was slowly added to the sample to precipitate the proteins, followed by vortex mixing and incubation at 4 °C for 2 h. The precipitated proteins were collected by centrifugation at 4500× *g* for 5 min at 4 °C. Subsequently, the collected precipitated proteins were washed with pre-cooled acetone three times and allowed to dry for 1 min. The washed and dried proteins were then redissolved in 200 mM TEAB and dispersed by ultrasonication. For the first digestion, trypsin was added at a 1:50 trypsin mass ratio overnight. Subsequently, the peptides were reduced with 5 mM dithiothreitol for 60 min at 37 °C, followed by 11 mM iodoacetamide for 45 min at room temperature and in the dark. Finally, the peptides were desalted, using a Strata X SPE column.

#### 2.3.3. Affinity Enrichment

Tryptic peptides were dissolved in NETN buffer (100 mM NaCl, 1 mM EDTA, 50 mM Tris-HCl, 0.5% NP-40, pH 8.0) to dissolve Kac-modified peptides. Pre-washed antibody beads (PTM-104, 10002D) were then added and incubated with the peptide mixture at 4 °C for an overnight period with gentle shaking. The beads were then washed twice with water and four times with NETN buffer. The use of 0.1% trifluoroacetic acid allowed the bound peptides to be released from the beads. The eluted portions were then mixed and dried under a vacuum. The resultant peptides were desalted using C18 ZipTips (ZTC18S) in accordance with the manufacturer’s instructions for LC-MS/MS analysis.

#### 2.3.4. LC-MS/MS Analysis and Database Search

Solvent A (0.1% formic acid, 2% acetonitrile/in water) was used to solubilize the tryptic peptides, which were then loaded directly onto a home-made analytical column (25-cm length, 100 μm i.d.). In a nanoElute UHPLC system (IQLAAAGABHFAPUMZZZ) running at 350 nL/min, the solvent B gradient increased from 6% to 22% (0.1% formic acid dissolved in acetonitrile) over 42 min, increased from 22% to 30% in 12 min, increased to 80% in 3 min, maintained at 80%, and then hold at 80% for 3 min. Prior to mass spectrometry analysis with timsTOF Pro, the peptides were subjected to Capillary source treatment. Then, 1.75 kV of electrospray voltage was used. At the TOF detector, precursors and fragments were analyzed using MS/MS with a scan range of 100 to 1700 *m*/*z*. Parallel accumulation serial fragmentation (PASEF) mode was used to run the timsTOF Pro. Ten PASEF-MS/MS scans were acquired per acquisition cycle, with the fragmentation of precursors with charge states 0 to 5 being the preferred range. A 24 s dynamic exclusion was set. The MaxQuant search engine (v. 1.6.6.0) was used to process the generated MS/MS data. Up to 4 missing cleavage events were made possible by using the cleavage protein trypsin/P. In the first search and the main search, the mass tolerance for precursor ions was set to 20 ppm and 20 ppm, respectively. And the mass tolerance for fragment ions was set as 20 ppm. Protein N-terminal acetylation, oxidation on Met, and acetylation on Lys were designated as variable modifications, while carbamidomethyl on Cys was designated as a fixed modification. FDR was adjusted to <1%.

#### 2.3.5. Bioinformatics Analysis

Firstly, the average quantification of the number of multiple repetitions in the CG and BG groups was first counted separately, and then the ratio of the average of the CG and BG groups was computed. This ratio was used for final quantification. Quantitative values were log-transformed and *p*-values were calculated from two-tailed Student’s *t*-tests. Significant differences were defined as protein or modified peptide *p* < 0.05, fold change (FC) ≥ 1.5. Functional annotation tools were used to identify enriched clusters of acetylated proteins.

GO enrichment analysis: protein GO annotations are classified into 3 categories: biological process, cellular component, and molecular function. The Fisher’s exact test was used to determine the significance of GO enrichment of different changed proteins, and the significance was *p*-value < 0.05.

KEGG pathway enrichment analysis: for the enrichment analysis of the KEGG pathway, we used the KEGG database. Fisher’s exact test was used to determine the significance of KEGG pathway enrichment of different changed proteins, and proteins were considered to be significant at a *p*-value of less than 0.05.

Protein subcellular localization: in this research, the subcellular localization of proteins is predicted using Wolfpsort(About WoLF PSORT (hgc.jp)) subcellular localization prediction software. Wolfpsort is applied to eukaryotic sequence prediction.

Protein motif analysis: by counting the pattern of amino acid sequences before and after all acetylation modification sites, a trend in the pattern of amino acid sequences within the region of the site where the modification occurs is calculated.

### 2.4. Modeling Acetylated Proteins Homology

To understand the structure of the protein and the level of acetylation modification sites, we used NCBI BLAST to find and compare homologous sequences. Subsequently, using SWISS-MODEL, a three-dimensional structural model of the protein was generated, labeling the acetylation sites.

### 2.5. Statistics and Analysis of Data

Chlorophyll fluorescence parameters measured in CG and BG groups were analyzed and plotted using SPSS Statistics 26.

## 3. Results

### 3.1. Quantitative Proteomic Analysis of Acetylated Modification in R. chrysanthum

In order to map the sites of lysine acetylation in *R. chrysanthum*, trypsin was used to break down proteins into peptides. *R. chrysanthum* was divided into two groups based on the treatments: the BG group (*R. chrysanthum* treated with PAR+UV−B for 48 h, three biological replicates) and the CG group (*R. chrysanthum* treated with PAR for 48 h, three biological replicates) groups. Then, an LC-MS/MS analysis was carried out (Supplementary Figure S1B). The majority of the acetylation-containing polypeptides were found to have lengths between 7 and 20, which is consistent with the fundamental principle of trypsin enzymatic digestion (Supplementary Figure S1C). A total of 945 lysine acetylation sites were present in 685 differentially expressed acetylated proteins (DAPs) (Supplementary Table S1). A total of 807 differentially expressed proteins (DEPs) were found using quantitative proteomics analysis; according to fold change >1.5 and *p* < 0.05, 357 of these proteins were downregulated and 450 were upregulated (Supplementary Table S2).

### 3.2. Plant Carbon Assimilation in R. chrysanthum Is Impacted by Photodamage under UV−B Stress

**Figure 1:**
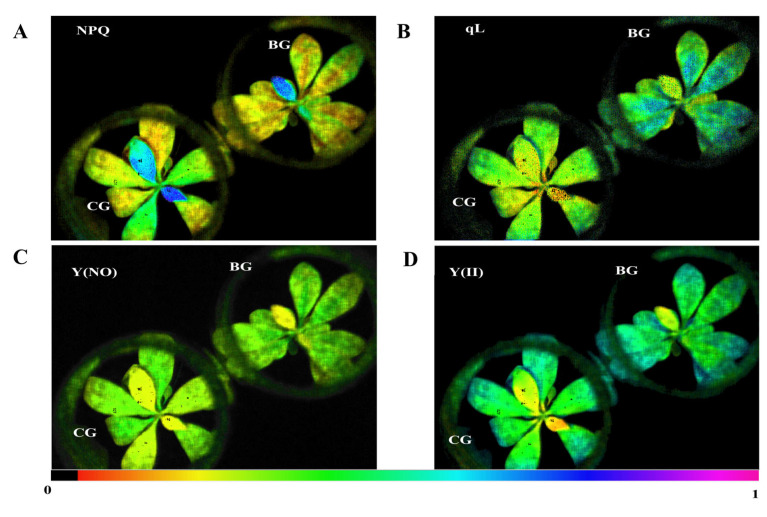
Imaging of real-time fluorescence of *R. chrysanthum* under UV−B stress. (**A**) average nonphotochemical quenching (NPQ); (**B**) photochemical quenching coefficient (qL)-based; (**C**) nonphotochemical quenching quantum yield Y(NO); and (**D**) actual photochemical quantum yield Y(II) for PSII. The color in the color scale of the picture indicates the size of the value, the more reddish the color indicates the smaller the value, and the more purple the color indicates the larger the value.

With the use of chlorophyll fluorescence technology, changes in leaf photosynthesis can be swiftly and precisely reflected. Chlorophyll fluorescence characteristics shift in plants exposed to UV−B stress, which in turn indicates photosynthetic damage. We determined the chlorophyll fluorescence parameters in order to investigate variations in *R. chrysanthum’s* photochemical activity. For pictures of chlorophyll fluorescence, see Figure 1. The plant’s NPQ is represented by the fluorescence images, which show a decline in *R. chrysanthum’s* ability to protect against photodamage (Figure 1A). The plant’s photosynthetic activity is reflected in qL, and under UV−B stress, *R. chrysanthum’s* photosynthetic activity increases as shown by the fluorescence images (Figure 1B). Additionally, the Y(NO) image shows that the plant has photodamaged (Figure 1C). The (YII) images show a decrease in *R. chrysanthum’s* actual photosynthetic rate. (Figure 1D).

**Figure 2:**
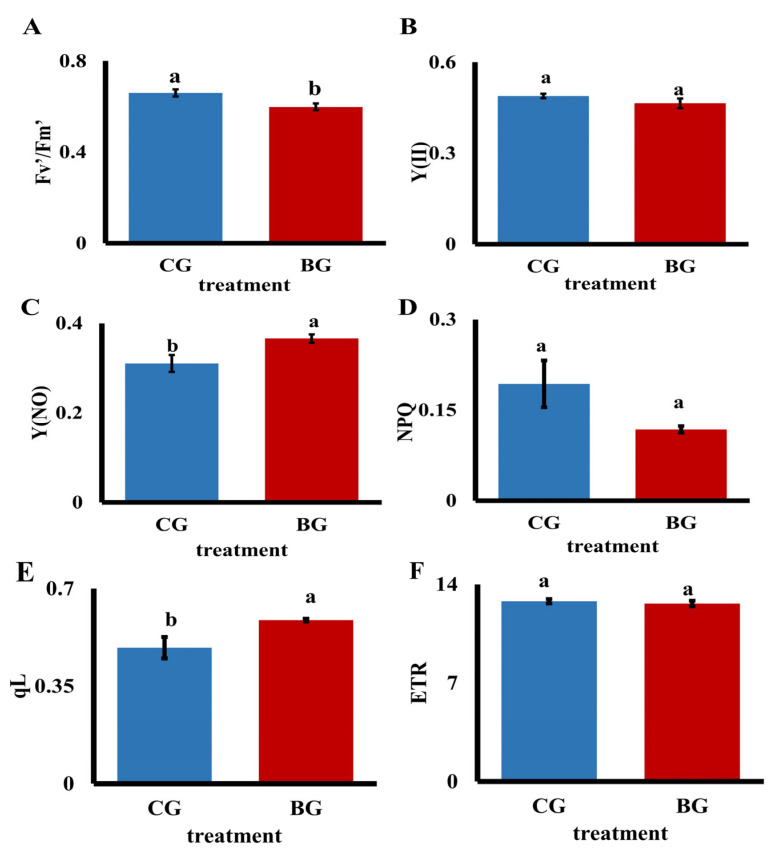
Chlorophyll fluorescence parameters in *R. chrysanthum*. a and b denote significant differences between PAR and PAR+UV−B (*p* < 0.05). (**A**) Maximum quantum yield of PSII (Fv′/Fm′); (**B**) Actual photochemical quantum yield of PSII [Y(II)]; (**C**) Non-photochemical quenching quantum yield [Y(NO)]; (**D**) Non-photochemical quenching coefficient (NPQ); (**E**) Photochemical quenching coefficient (qL); (**F**) Electron transfer rate (ETR). These results are the mean ± standard deviation of three samples.

The rate of Fv′/Fm′ significantly dropped by 9.25% under UV−B stress (Figure 2A, Table 1), suggesting that photoinhibition was taking place and damaging the reaction center of chrysanthemum photosystem II. The real light energy conversion efficiency of leaves over time is represented by Y(II) (Figure 2B, Table 1). However, Y(NO), the quantum yield of non-photochemical quenching, is a crucial marker of photodamage, and the analysis reveals a notable 15.2% increase in Y(NO) value. The plant damage caused by UV−B radiation was indicated by the significant increase in Y(NO) value (Figure 2C, Table 1). The ability of the plant to transform excess light energy into heat energy is reflected in non-photochemical quenching, which shields the plant from harm. The plant’s capacity to disperse heat is indicated by NPQ. The plant’s capacity to disperse heat is indicated by NPQ (Figure 2D, Table 1). Photochemical quenching reflects the photosynthetic activity of the plant, while non-photochemical quenching represents the ability of the plant to convert excess light energy into heat. Under UV−B stress, the qL of *R. chrysanthum* increased by 20.4%. This indicates that the photosynthetic activity of *R. chrysanthum* increased under UV−B stress (Figure 2E, Table 1). The rate of electron transfer can serve as an indicator of the photosynthesis’s rate of electron transfer. *R. chrysanthum’s* electron transfer rate (ETR) values exhibited a declining trend (Figure 2F, Table 1), suggesting that UV−B stress could have an impact on the plant. It is a crucial component of the light response curve and can be used to estimate the rate at which electron transfer energy is converted.

### 3.3. Analysis of Distribution and Motif of Acetylated Sites in Photosynthetic Carbon Cycle of R. chrysanthum

**Figure 3:**
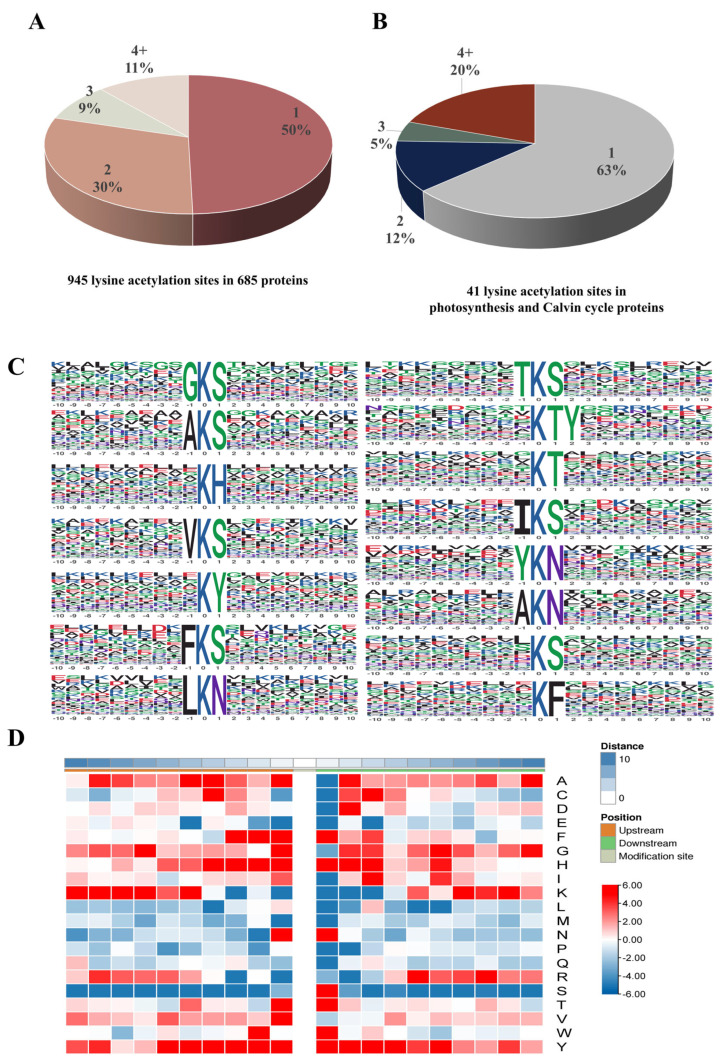
Properties of lysine acetylation sites in photosynthesis of *R. chrysanthum* under UV−B stress. (**A**) The number and proportion of lysine acetylation sites. (**B**) The number and percentage of lysine acetylationproteins sites in photosynthesis and Calvin cycle. (**C**) Sequence probability logos for acetylation site motifs significantly enriched for ± 10 amino acids around the lysine acetylation sites. (**D**) Clustering heatmap of amino acids up-stream and down-stream the acetylation modification site. Red color is that this amino acid is significantly enriched near the modification site, and blue is a significant decrease.

The number of detected modification sites per protein was estimated in order to evaluate the sites’ distribution in the acetylated proteins of *R. chrysanthum*. The findings suggested that 50% of proteins had only 1 site, whereas 30%, 9%, and 11% of proteins had 2, 3, or more modification sites, respectively (Figure 3A). Acetylated proteins are enriched in two processes: photosynthesis and the Calvin cycle. Only 63% of proteins had one acetylation site, whereas 12%, 5%, and 20%, respectively, of proteins had 2, 3, or more modification sites (Figure 3B). The sequence of amino acid distribution around the acetylated lysine was analyzed to comprehend the motifs in *R. chrysanthum*. Using motif-x, a total of 15 significantly enriched acetylation motifs were screened, of which the three motifs “KT”, “KY”, and “KS” accounted for the largest proportion. (Figure 3C, Supplementary Table S3). These results indicate that the conserved motifs and residues of the proteins in which *R. chrysanthum* undergoes acetylation are more intact and suggest that the lysine residues surrounding the basic residues in *R. chrysanthum* proteins have a greater chance of being acetylated under UV−B stress. The thermogram of compositions of amino acid around the acetylation sites showed that histidine (H) and tyrosine (Y) were apparently excessive from −4 to +4, and others, the cases in point are phenylalanine (F) and serine (S). They were highly enriched at +1 (Figure 3D, Supplementary Table S4).

### 3.4. Analysis of Functional Enrichment of Acetylated Proteins and Sites in Photosynthetic Carbon Cycle of R. chrysanthum

**Figure 4:**
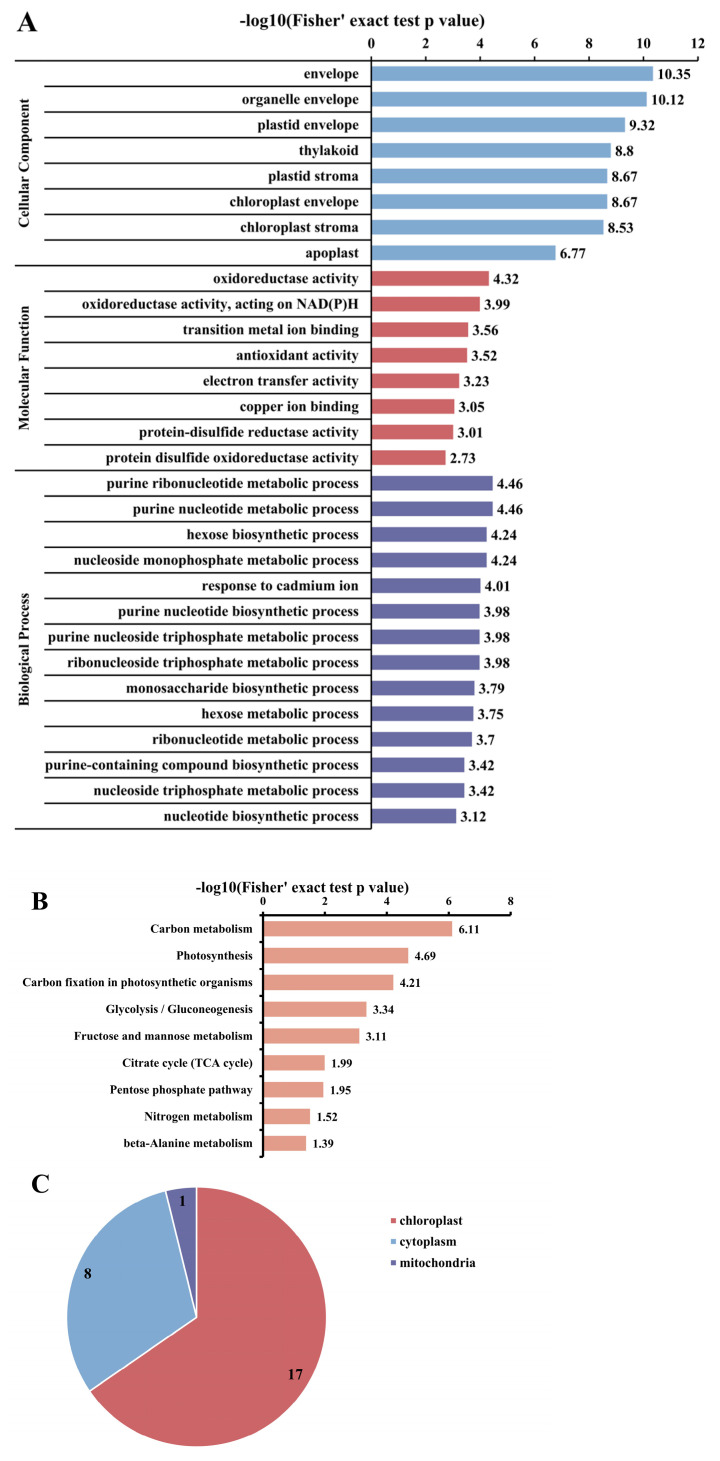
Enrichment analysis of the acetylated proteins in carbon fixation of *R. chrysanthum*. (**A**) The acetylated proteome is analyzed for GO enrichment. (**B**) The acetylated proteome is analyzed for KEGG pathway. (**C**) Subcellular localization of acetylated proteins of carbon fixation.

With the aim of further investigating the acetylated proteins, they were divided into 3 main categories: biological process, molecular function, and cellular component (Supplementary Table S5). We carried out GO enrichment (Figure 4A, Supplementary Tables S6–S8). According to the cellular component analysis, *R. chrysanthum*’s acetylation-modified proteins were dramatically enriched in metabolic processes, and proteins enriched in the envelope and chloroplast were more likely to be acetylated (Figure 4A). Acetylation-modified proteins from *R. chrysanthum* were enriched in KEGG metabolic pathways, which allows a better understanding of their general function (Figure 4B, Supplementary Table S9). The findings demonstrated that some proteins in carbon metabolism undergo lysine acetylation (Supplementary Figure S2), the process of photosynthesis (Supplementary Figure S3), as well as carbon fixation in photosynthetic organisms (Supplementary Figure S4). KEGG enrichment showed that 26 acetylated proteins were acetylated in the carbon fixation of photosynthetic organisms, and 53 acetylation sites were identified. Acetylated proteins of *R. chrysanthum* in carbon fixation are localized to chloroplasts, mitochondria, and cytoplasm, as shown by subcellular localization with 17, 1, and 8 (Figure 4C).

### 3.5. Analysis of DEPs and DAPs in Carbon Fixation in Photosynthetic Organisms of R. chrysanthum under UV−B Stress

**Figure 5:**
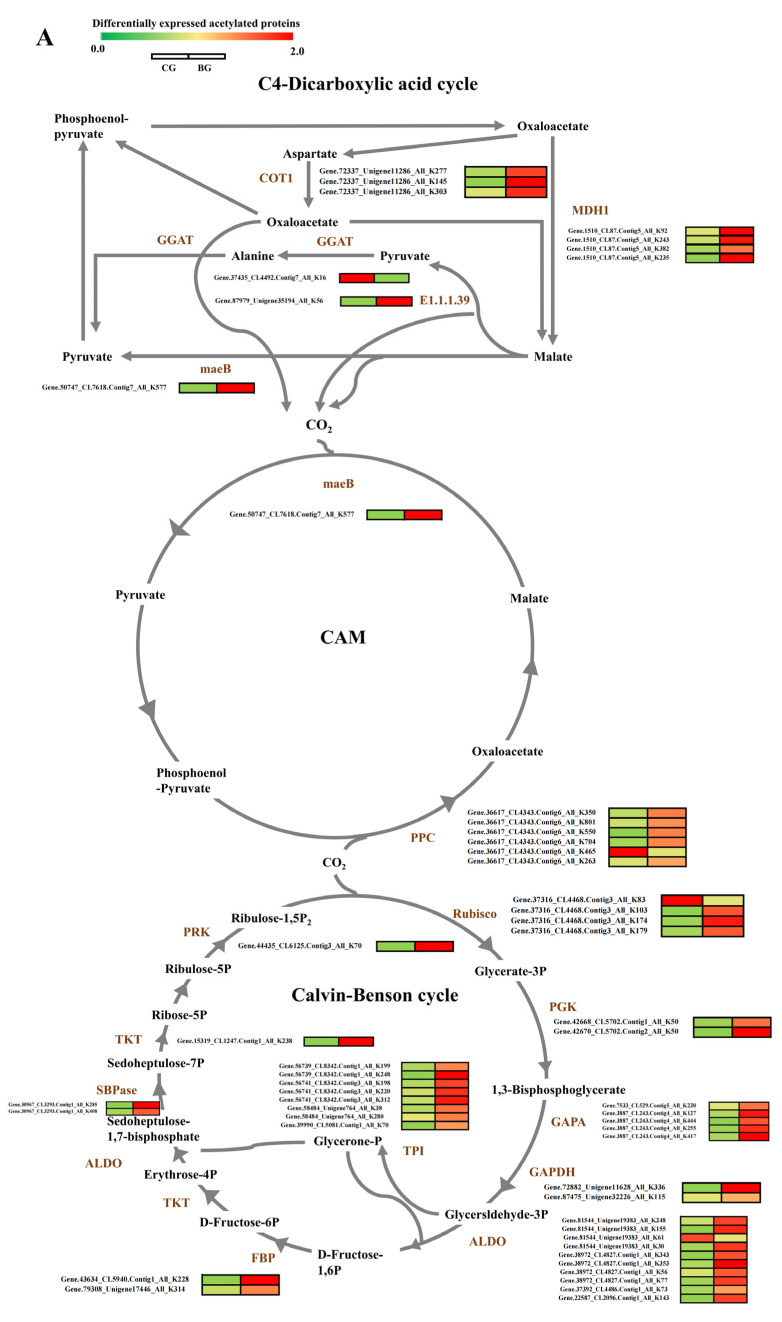

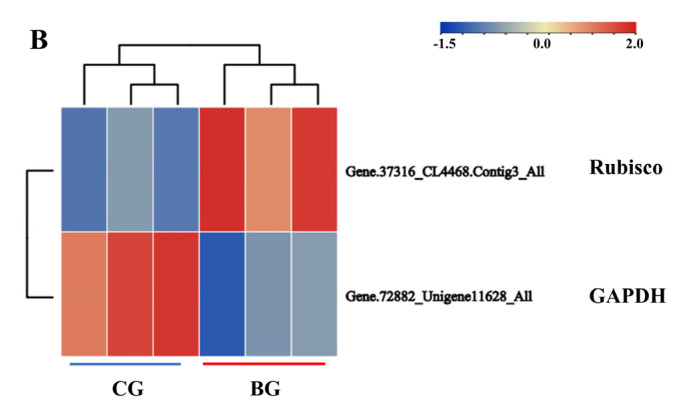
Working scheme of DAPs, sites and DEPs in carbon fixation in photosynthetic organisms in *R. chrysanthum*. (**A**) DAPs involved in carbon fixation in photosynthetic organisms. Rubisco: ribulose bisphosphate carboxylase/oxygenase; PGK: phosphoglycerate kinase; GAPDH: glyceraldehyde 3-phosphate dehydrogenase; GAPA: Glyceraldehyde-3-phosphate dehydrogenase B; ALDO: Fructose-bisphosphate aldolase 3; TPI: triosephosphate isomerase; FBP: fructose-1,6-bisphosphatase; TKT: Transketolase; SBPase: Sedoheputulose-1,7-bisphosphatase; PRK: phosphoribulokinase; PPC: Phosphoenolpyruvate carboxylase; maeB: NADP-dependent malic enzyme; MDH1: Malate dehydrogenase [NADP]; GGAT: Glutamate-glyoxylate aminotransferase 2; COT1: Aspartate aminotransferase; E1.1.1.39: malate dehydrogenase (decarboxylating). (**B**) Clustering heat map of DEPs in carbon fixation in photosynthetic organisms in the *R. chrysanthum* under UV−B stress.

Based on the outcomes of acetylated proteins’ functional enrichment analysis, it was shown that lysine acetylation has a significant role in the pathways of photosynthesis and carbon fixation of plants. We analyzed the proteins in the carbon fixation pathway and found that the enzymes for carbon fixation under UV−B stress underwent acetylation modification with different levels of modification at different sites (Figure 5A). Carbon fixation is a reaction in photosynthesis that does not require the participation of light and involves the conversion of carbon dioxide, as well as other compounds, into glucose through a series of reactions. In the analysis of data from *R. chrysanthum* in the Calvin–Benson cycle, we found up-regulation of acetylation modification levels at the sites of metabolic enzymes Rubisco, PGK, GAPA, GAPDH, ALDO, FBP, SBPase, PRK, and TKT. In CAM, the PPC and maeB enzymes of *R. chrysanthum* were modified by acetylation under UV−B stress, and the level of modification was up-regulated. In the C4-dicarboxylic acid cycle, we also identified the acetylated protein. In this pathway, acetylation-modified MDH1, COT1, GGAT, maeB and malate dehydrogenase (E1.1.1.39) were recognized, with reduced levels of acetylation modification only at the malate dehydrogenase site (Figure 5A). We further analyzed this by quantitative proteomics and found that the only proteins that underwent acetylation modification were Rubisco and GAPDH enzymes, which showed significant differential expression of the proteins. Rubisco protein expression was up-regulated in *R. chrysanthum* after UV−B stress, which was positively correlated with the level of acetylation modification. GAPDH protein expression was down-regulated and inversely proportional to the acetylation modification level (Figure 5B, Supplementary Table S2). Taken together, these results suggest that proteins undergo acetylation modification and that the level of acetylation modification has an impact on the expression of proteins, which in turn affects the processes of photosynthesis and carbon fixation in plants in response to UV−B stress.

### 3.6. Protein Structure Analysis of Rubisco and GAPDH Enzymes Undergoing Acetylation Modification in R. chrysanthum under UV−B Stress

**Figure 6:**
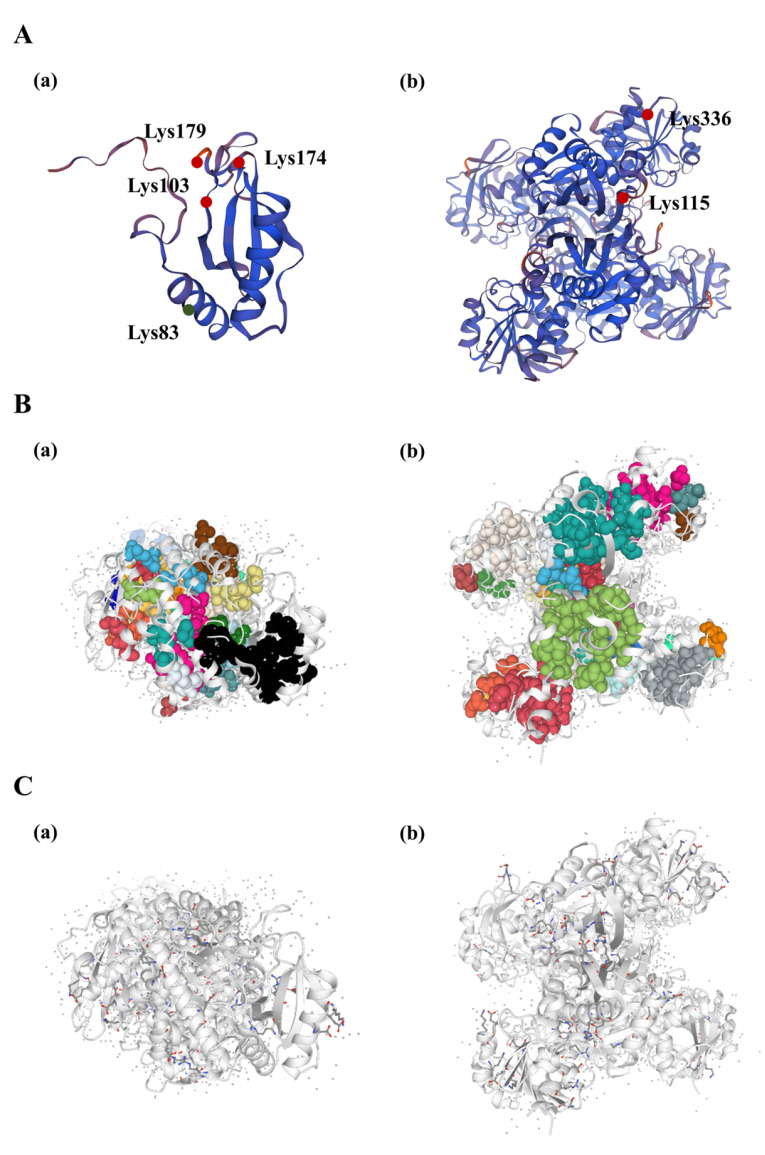
Three-dimensional structure, hydrophobic clusters and salt bridges of acetylation-modifying enzymes for carbon fixation in *R. chrysanthum* under UV−B stress. (**A**) Three-dimensional structure of acetylation-modifying enzymes for carbon fixation. Red is up-regulation of site acetylation modification levels, green is down-regulation of site acetylation modification levels (**B**) Hydrophobic clusters of acetylation-modifying enzymes for carbon fixation; (**C**) Salt bridges of acetylation-modifying enzymes for carbon fixation. (**a**) is Rubisco, (**b**) is GAPDH.

The level of protein acetylation in carbon fixation and photosynthesis of *R. chrysanthum* was shown to be higher throughout the entire period of UV−B stress (Supplementary Table S10). By acetylation modification proteomic analysis, we found that acetylation modification occurred mainly for the Rubisco enzyme and the GAPDH enzyme during carbon fixation after UV−B radiation, and significant differences in expression occurred after modification. Therefore, we concluded that these two enzymes play important roles in the carbon fixation process of *R. chrysanthum* under UV−B stress. In order to understand the features and functions of Rubisco and GAPDH proteins at the molecular level, we described the hydrophobic clusters and salt bridges of the proteins. Firstly, we constructed three-dimensional models of the proteins. We used SWISS-MODEL to build statistically acceptable homology models. The acetylation sites were labelled in the 3D structure based on the information on the acetylation sites (Figure 6A). In Rubisco proteins, only the acetylation level of the Lys83 site was down-regulated, and the Lys103, Lys174, and Lys174 sites were up-regulated. In GAPDH proteins, both Lys115 and Lys336 sites were upregulated (Figure 6A(a,b)). The hydrophobic structure was analyzed, and the total area of the largest hydrophobic cluster of the GAPDH protein was 3798.7^2^, with a single cluster containing 22 residues. The area of each residue is 42.7^2^ and there are 89 interactions between residues (Figure 6B(a), Supplementary Table S11). In Rubisco, the total area of the largest hydrophobic cluster of the Rubisco protein was 1657.1^2^, with a single cluster containing 16 residues. The area of each residue is 41.4^2^ and there are 40 interactions between residues (Figure 6B(b), Supplementary Table S10). By calculating the charge separation parameters, we can obtain the fraction of charged residues (FCR) of GAPDH as 0.24 and Kappa value (K) as 0.16. The FCR of Rubisco is 0.24 and K is 0.14 (Figure 6C(a,b), Supplementary Table S11).

## 4. Discussion

Lysine acetylation of proteins plays an important regulatory role in plants with different biological functions. So far, acetylated proteins have been reported only in some plants, including *Arabidopsis* [27], wheat [28], poplar [21], rice [29], and pepper [30]. *R. chrysanthum* is a valuable species resource that can withstand UV−B exposure due to its unique growth environment [1]. As a result, we investigated the lysine acetylation proteomics of *R. chrysanthum* under UV−B stress, using *R. chrysanthum* as experimental material. In total, 685 acetylated proteins and 945 modification sites were found using acetylation proteomics analysis. In the natural world, photosynthesis is how plants create the organic materials needed for their own growth and development [30]. However, important enzymes involved in plant carbon fixation are somewhat impacted by abiotic stress, which has an impact on the quantity of CO_2_ fixation [31]. In order to identify the mechanism underlying *R. chrysanthum*’s resistance to UV−B light, these acetylated proteins were categorized based on their GO function and examined with a focus on the acetylation of proteins during carbon fixation.

As the physiological process that provides plants with their energy, photosynthesis is crucial to the functioning of plants. Under UV−B radiation, many plants usually have obvious stress responses, resulting in damage such as inactivation of PSII reaction centers or enhanced photoinhibition [32,33]. Light plays a crucial role in photosynthesis, and the light reaction produces ATP and NADPH to drive the dark reaction. The photosynthetic system of plants utilizes light primarily through photosynthetic pigments in the membranes of the cysts [30]. Photosystem II decomposes H_2_O, produces protons and releases O_2_ through absorbed light energy. But, UV−B destroys PSII but has no direct effect on PSI. Nevertheless, PSI and PSII drive photosynthetic electron transport sequentially. A study demonstrates that UV−B improves chilling-light-induced PSI photoinhibition and promotes CO_2_ assimilation recovery in cucumber (*Cucumis sativus* L.) [34]. Chlorophyll fluorescence parameters are useful indicators of photosynthesis. The analysis of various fluorescence parameters can reveal a number of regulatory processes within photosynthetic mechanisms. Fluorescence parameters such as Fv/Fm and Y(II) are usually reduced in plants under stress conditions [35,36]. In this study, the results of chlorophyll fluorescence parameters of *R. chrysanthum* under UV−B radiation treatment showed that Y(II) was significantly reduced compared to the control (Figure 2). This is consistent with the research results of UV−B radiation on water moss (*Fontinalis antipyretica* Hedw.) [37], indicating that under UV−B radiation stress, the PSII reaction center of plants is significantly damaged, leading to the occurrence of photoinhibition, which in turn affects photosynthetic activity. In addition, to explore the photodamage condition of *R. chrysanthum* under UV−B stress, we analyzed more chlorophyll fluorescence parameters such as NPQ, qL, ETR. Taken together, these results indicate that UV−B can cause some damage to the plant’s photosynthetic system, but the plant itself responds to this damage through a series of reactions. To further investigate the molecular mechanisms of plant resistance to UV−B stress, we performed proteomic and acetylation modification analyses.

These amino acids interact and function collectively rather than separately. Since lysine’s acetylation modification may have an impact on nearby amino acids, which may also play certain physiological roles in conjunction with lysine, the sequences of lysines undergoing acetylation modification and their neighboring amino acids will be shown in this study (Figure 3). More research is necessary to determine how nearby amino acids are affected by lysine acetylation modification and how lysine acetylation modification functions in conjunction with them. Hydrophobic structures and salt bridges are crucial for preserving the steric structure of proteins (Figure 6). Proteins’ three-dimensional structures determine their functions, so in order to better understand how these proteins behave under UV−B stress, their salt-bridge and hydrophobic structures were further characterized after the target’s protein structure was established.

Proteins ensure normal plant metabolism, and some proteins (e.g., Rubisco) play an important role in various physiological metabolic processes as biocatalytic enzymes, whose maximum absorption wavelengths fall within the wavelength range of UV−B radiation. Acetylated modification can produce a range of biochemical and physiological reactions [38]. In chloroplasts, in the Calvin cycle, GAPDH plays a crucial role by specifically binding with NADPH [39]. By shaping an invertible multi-enzyme complex with CP12, GAPDH and PRK commonly regulate and control the Calvin cycle [40]. Rubisco, a protein in the chloroplast stroma that catalyzes the carboxylation of ribulose-1,5-bisphosphate for CO_2_ fixation, is an important enzyme in the Calvin cycle’s first step. In plants, carbon enters the Calvin cycle in the form of carbon dioxide, so enzymes play a crucial role in the Calvin cycle process. In the study, in the Calvin cycle process, the key enzymes were acetylated in *R. chrysanthum* (Figure 5A). This is consistent with previous findings that acetylation modifications of enzymes involved in the Calvin cycle process in the face of abiotic stresses were similarly found in pepper and wheat [28,30]. Interestingly, ribulose-diphosphate carboxylase (Rubisco) contains 4 acetylation sites in *R. chrysanthum* (Supplemental Table S9). In fact, the carbon fixation of plants is closely related to environmental factors. High UV−B radiation reduced carbon fixation rates in plants [41]. Our results suggest that under UV−B stress, plant photosystems are impaired and photosynthesis is altered, and thus the carbon assimilation process is inevitably affected to some extent. However, the level of acetylation modification affects the expression of plant proteins, which in turn adjusts the alterations that occur in the carbon assimilation process.

**Figure 7:**
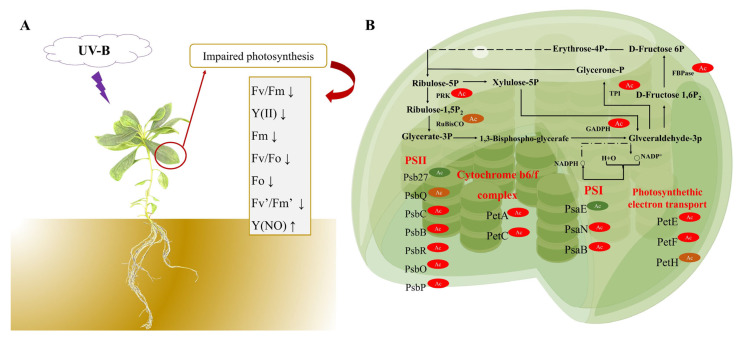
Mechanism diagram of *R. chrysanthum*’s photosynthesis resistance to UV−B stress. (**A**) Altered photosynthesis in *R. chrysanthum* under UV−B stress. (**B**) Schematic diagram of photosynthesis and carbon fixation acetylation of *R. chrysanthum* under UV−B stress. Upward arrows indicate elevated levels, and downward arrows indicate decreased levels.

In our study, photosynthesis of *R. chrysanthum* was inhibited by UV−B radiation with a significant increase in qL and a significant decrease in NPQ by biochemical analyses, indicating that the photosystem was damaged. The acetylation proteome reveals that the photosynthetic proteins undergo acetylation modification, while the level of key acetylation modification enzymes in the pathway of the carbon cycle shows an upward trend (Figure 7).

## 5. Conclusions

In conclusion, we identified 685 proteins and discovered 945 acetylation modifications at their sites using acetylation proteomics data analysis. Further GO analysis revealed that these proteins are involved in a diverse set of biological processes. According to functional enrichment analysis, acetylated proteins play a role in *R. chrysanthum* photosynthesis and carbon fixation. It was discovered that acetylation modification of key Calvin cycle enzymes (Rubisco, GAPDH) modulates protein expression, causing Rubisco and GAPDH proteins to be expressed in significantly different ways, affecting *R. chrysanthum*’s carbon fixation capacity. As a result of the acetylation modification, Rubisco and GAPDH are significantly differentially expressed, affecting carbon fixation capacity and making the plant resistant to UV−B stress. This study provides valuable information about the plant’s response to UV−B and lays the groundwork for future protein post-translational modification research.

## Figures and Tables

**Table 1:** Effect of UV−B stress on chlorophyll fluorescence parameters of *R. chrysanthum*.

Group	Fv′/Fm′	Y(II)	Y(NO)	NPQ	qL	ETR
CG	0.660 ± 0.015a	0.489 ± 0.008a	0.310 ± 0.019b	0.193 ± 0.039a	0.49 ± 0.038b	12.8 ± 0.163a
BG	0.599 ± 0.014b	0.465 ± 0.016a	0.366 ± 0.009a	0.118 ± 0.006a	0.59 ± 0.005a	12.6 ± 0.205a

Note: These results represent the average ± standard deviation of 3 samples. Different lowercase letters suggest significant inter-group differences (*p* < 0.05), whereas identical lower-case characters suggest no relevant difference across groups.

## Data Availability

The datasets generated during and/or analysed during the current study are available from the corresponding author on reasonable request.

## Supplementary Materials

The following supporting information can be downloaded at: https://www.mdpi.com/article/10.3390/biom14060732/s1, Figure S1: Identification of lysine acetylation proteome in *Rhododendron chrysanthum*. (A) 4D-label free experimental flowchart. (B) Mass error distribution of whole identified polypeptides. (C) The length distribution of identified peptide; Figure S2. KEGG pathway enrichment analysis of the acetylated proteins in carbon metabolism. The acetylated proteins are in blue; Figure S3. KEGG pathway enrichment analysis of the acetylated proteins in photosynthesis. The acetylated proteins are in blue; Figure S4. KEGG pathway enrichment analysis of the acetylated proteins in carbon fixation in photosynthetic organisms. The acetylated proteins are in blue; Table S1. The identified acetylated sites in *Rhododendron chrysanthum*; Table S2. The identified differentially expressed proteins in *Rhododendron chrysanthum*; Table S3. Peptide motif of *R. chrysanthum*; Table S4. Frequency of the different types of amino acids around the acetylated lysine; Table S5. Go annotation detail of the acetylated proteins in *Rhododendron chrysanthum*; Table S6. Protein GO enrichment in biological process; Table S7. Protein GO enrichment in molecular function; Table S8. Protein GO enrichment in cellular component; Table S9. Protein pathway enrichment; Table S10. Differential acetylated proteins *Rhododendron chrysanthum* under UV−B stress; Table S11. Structural information on hydrophobic clusters and salt bridges in *R. chrysanthum* under UV−B stress.

## References

[1] Zhou X., Chen S., Wu H., Yang Y., Xu H. (2017). Biochemical and proteomics analyses of antioxidant enzymes reveal the potential stress tolerance in *Rhododendron chrysanthum* Pall. Biol. Direct..

[2] Lyu J., Wang C., Liang D.Y., Liu L., Pandey L.K., Xu H.W., Zhou X.F. (2019). Sensitivity of wild and domesticated Rhododendron chrysanthum to different light regime (UVA, UVB, and PAR). Photosynthetica.

[3] Bassman J.H. (2004). Ecosystem consequences of enhanced solar ultraviolet radiation: Secondary plant metabolites as mediators of multiple trophic interactions in terrestrial plant communities. Photochem. Photobiol..

[4] Knox P.P., Lukashev E.P., Gorokhov V.V., Grishanova N.P., Paschenko V.Z. (2019). Hybrid complexes of photosynthetic reaction centers and quantum dots in various matrices: Resistance to UV irradiation and heating. Photosynth. Res..

[5] Widel M., Krzywon A., Gajda K., Skonieczna M., Rzeszowska-Wolny J. (2014). Induction of bystander effects by UVA, UVB, and UVC radiation in human fibroblasts and the implication of reactive oxygen species. Free Radic. Biol. Med..

[6] Hideg E., Jansen M.A., Strid A. (2013). UV−B exposure, ROS, and stress: Inseparable companions or loosely linked associates?. Trends Plant Sci..

[7] Chen Y.-E., Su Y.-Q., Zhang C.-M., Ma J., Mao H.-T., Yang Z.-H., Yuan M., Zhang Z.-W., Yuan S., Zhang H.-Y. (2018). Comparison of Photosynthetic Characteristics and Antioxidant Systems in Different Wheat Strains. J. Plant Growth Regul..

[8] Barber J. (1998). Photosystem two. Biochim. Biophys. Acta Bioenerg..

[9] Yoon H.I., Kim D., Son J.E. (2020). Spatial and Temporal Bioactive Compound Contents and Chlorophyll Fluorescence of Kale (*Brassica oleracea* L.) Under UV−B Exposure Near Harvest Time in Controlled Environments. Photochem. Photobiol..

[10] Piccini C., Cai G., Dias M.C., Romi M., Longo R., Cantini C. (2020). UV−B Radiation Affects Photosynthesis-Related Processes of Two Italian *Olea europaea* (L.) Varieties Differently. Plants.

[11] Liu M., Sun Q., Cao K., Xu H., Zhou X. (2023). Acetylated Proteomics of UV−B Stress-Responsive in Photosystem II of *Rhododendron chrysanthum*. Cells.

[12] UniProt Consortium T. (2018). UniProt: The universal protein knowledgebase. Nucleic Acids Res..

[13] Choudhary C., Weinert B.T., Nishida Y., Verdin E., Mann M. (2014). The growing landscape of lysine acetylation links metabolism and cell signalling. Nat. Rev. Mol. Cell Biol..

[14] Narita T., Weinert B.T., Choudhary C. (2019). Functions and mechanisms of non-histone protein acetylation. Nat. Rev. Mol. Cell Biol..

[15] Colville A., Alhattab R., Hu M., Labbé H., Xing T., Miki B. (2011). Role of HD2 genes in seed germination and early seedling growth in Arabidopsis. Plant Cell Rep..

[16] Kim W., Latrasse D., Servet C., Zhou D.X. (2013). Arabidopsis histone deacetylase HDA9 regulates flowering time through repression of AGL19. Biochem. Biophys. Res. Commun..

[17] Luo M., Wang Y.Y., Liu X., Yang S., Lu Q., Cui Y., Wu K. (2012). HD2C interacts with HDA6 and is involved in ABA and salt stress response in Arabidopsis. J. Exp. Bot..

[18] Park H.J., Baek D., Cha J.Y., Liao X., Kang S.H., McClung C.R., Lee S.Y., Yun D.J., Kim W.Y. (2019). HOS15 Interacts with the Histone Deacetylase HDA9 and the Evening Complex to Epigenetically Regulate the Floral Activator GIGANTEA. Plant Cell.

[19] van Zanten M., Zöll C., Wang Z., Philipp C., Carles A., Li Y., Kornet N.G., Liu Y., Soppe W.J. (2014). HISTONE DEACETYLASE 9 represses seedling traits in Arabidopsis thaliana dry seeds. Plant J..

[20] Yuan L., Chen X., Chen H., Wu K., Huang S. (2019). Histone deacetylases HDA6 and HDA9 coordinately regulate valve cell elongation through affecting auxin signaling in Arabidopsis. Biochem. Biophys. Res. Commun..

[21] Liao X., Li Y., Hu Z., Lin Y., Zheng B., Ding J. (2021). Poplar acetylome profiling reveals lysine acetylation dynamics in seasonal bud dormancy release. Plant Cell Environ..

[22] Zhu G.R., Yan X., Zhu D., Deng X., Wu J.S., Xia J., Yan Y.M. (2018). Lysine acetylproteome profiling under water deficit reveals key acetylated proteins involved in wheat grain development and starch biosynthesis. J. Proteom..

[23] Gong X., Huang Y., Liang Y., Yuan Y., Liu Y., Han T., Li S., Gao H., Lv B., Huang X. (2022). OsHYPK-mediated protein N-terminal acetylation coordinates plant development and abiotic stress responses in rice. Mol. Plant.

[24] Du Q., Qu Z., Wang L., Jiang J., Fu X., Fang Y., Li X., Xie X. (2021). Histone deacetylase SbHDT701 in Sorghum bicolor reveals functions in response to stress factors by enhancing acetylation. Pestic. Biochem. Physiol..

[25] Wu X., Oh M.H., Schwarz E.M., Larue C.T., Sivaguru M., Imai B.S., Yau P.M., Ort D.R., Huber S.C. (2011). Lysine acetylation is a widespread protein modification for diverse proteins in Arabidopsis. Plant Physiol..

[26] Jiang J., Gai Z., Wang Y., Fan K., Sun L., Wang H., Ding Z. (2018). Comprehensive proteome analyses of lysine acetylation in tea leaves by sensing nitrogen nutrition. BMC Genom..

[27] König A.C., Hartl M., Boersema P.J., Mann M., Finkemeier I. (2014). The mitochondrial lysine acetylome of *Arabidopsis*. Mitochondrion.

[28] Zhang Y., Song L., Liang W., Mu P., Wang S., Lin Q. (2016). Comprehensive profiling of lysine acetylproteome analysis reveals diverse functions of lysine acetylation in common wheat. Sci. Rep..

[29] Nallamilli B.R., Edelmann M.J., Zhong X., Tan F., Mujahid H., Zhang J., Nanduri B., Peng Z. (2014). Global analysis of lysine acetylation suggests the involvement of protein acetylation in diverse biological processes in rice (*Oryza sativa*). PLoS ONE.

[30] Liu Z., Song J., Miao W., Yang B., Zhang Z., Chen W., Tan F., Suo H., Dai X., Zou X. (2021). Comprehensive Proteome and Lysine Acetylome Analysis Reveals the Widespread Involvement of Acetylation in Cold Resistance of Pepper (*Capsicum annuum* L.). Front. Plant Sci..

[31] Savitch L.V., Leonardos E.D., Krol M., Jansson S., Öquist G. (2002). Two different strategies for light utilization in photosynthesis in relation to growth and cold acclimation. Plant Cell Environ..

[32] Wu J.-b., Guan D.-x., Yuan F.-h., Zhang X.-j. (2009). Research advances on the biological effects of elevated ultraviolet-B radiation on terrestrial plants. J. For. Res..

[33] Lidon F.J.C. (2012). Micronutrients’ accumulation in rice after supplemental UV−B irradiation. J. Plant Interact..

[34] Zhang Z.S., Jin L.Q., Li Y.T., Tikkanen M., Li Q.M., Ai X.Z., Gao H.Y. (2016). Ultraviolet−B Radiation (UV−B) Relieves Chilling-Light-Induced PSI Photoinhibition And Accelerates The Recovery Of CO_2_ Assimilation In Cucumber (*Cucumis sativus* L.) Leaves. Sci. Rep..

[35] Liang Y., Chen H., Tang M.J., Yang P.F., Shen S.H. (2007). Responses of Jatropha curcas seedlings to cold stress: Photosynthesis-related proteins and chlorophyll fluorescence characteristics. Physiol. Plant..

[36] Belkhodja R., Morales F., Abadia A., Gomez-Aparisi J., Abadia J. (1994). Chlorophyll Fluorescence as a Possible Tool for Salinity Tolerance Screening in Barley (*Hordeum vulgare* L.). Plant Physiol..

[37] Martínez-Abaigar J., Núñez-Olivera E., Beaucourt N., García-Álvaro M.A., Tomás R., Arróniz M. (2003). Different physiological responses of two aquatic bryophytes to enhanced ultraviolet−B radiation. J. Bryol..

[38] Ifuku K., Ido K., Sato F. (2011). Molecular functions of PsbP and PsbQ proteins in the photosystem II supercomplex. J. Photochem. Photobiol. B Biol..

[39] McFarlane C.R., Shah N.R., Kabasakal B.V., Echeverria B., Cotton C.A.R., Bubeck D., Murray J.W. (2019). Structural basis of light-induced redox regulation in the Calvin-Benson cycle in cyanobacteria. Proc. Natl. Acad. Sci. USA.

[40] Howard T.P., Lloyd J.C., Raines C.A. (2011). Inter-species variation in the oligomeric states of the higher plant Calvin cycle enzymes glyceraldehyde-3-phosphate dehydrogenase and phosphoribulokinase. J. Exp. Bot..

[41] Martínez-Lüscher J., Morales F., Sánchez-Díaz M., Delrot S., Aguirreolea J., Gomès E., Pascual I. (2015). Climate change conditions (elevated CO_2_ and temperature) and UV−B radiation affect grapevine (*Vitis vinifera* cv. Tempranillo) leaf carbon assimilation, altering fruit ripening rates. Plant Sci..

