# Supplementary material for: The Rhododendron Chrysanthum Pall.s’ Acetylation Modification of Rubisco Enzymes Controls Carbon Cycling to Withstand UV−B Stress
Source: Biomolecules. 2024 Jun 20;14(6):732. doi: 10.3390/biom14060732 (PMC11201758; doi:10.3390/biom14060732)
Supplement: Supplementary file 1 [file biomolecules-14-00732-s001.zip › Supplementary File/biomolecules-3033990-supplementary.pdf]

Supplementary Material

Acetylation modification of Rubisco and GAPDH enzymes in the *Rhododendron chrysanthum* Pall. regulates carbon cycling to resist UV-B stress

Meiqi Liu[1,†], Xiangqun Li[2,†], He Qi[1], Li Sun[3], Hongwei Xu[1] and Xiaofu Zhou[1,*]

[1]Jilin Provincial Key Laboratory of Plant Resource Science and Green Production, Jilin Normal University, Siping, China.

[2]Jilin Engineering Vocational College, Siping, China.

[3] Siping Central People's Hospital, Siping, China.

† These authors contributed equally to this work and share first authorship.

*** Correspondence:** Xiaofu Zhou zhouxiaofu@jlnu.edu.cn.

1 Supplementary Figures and Tables

1.1 Supplementary Figures

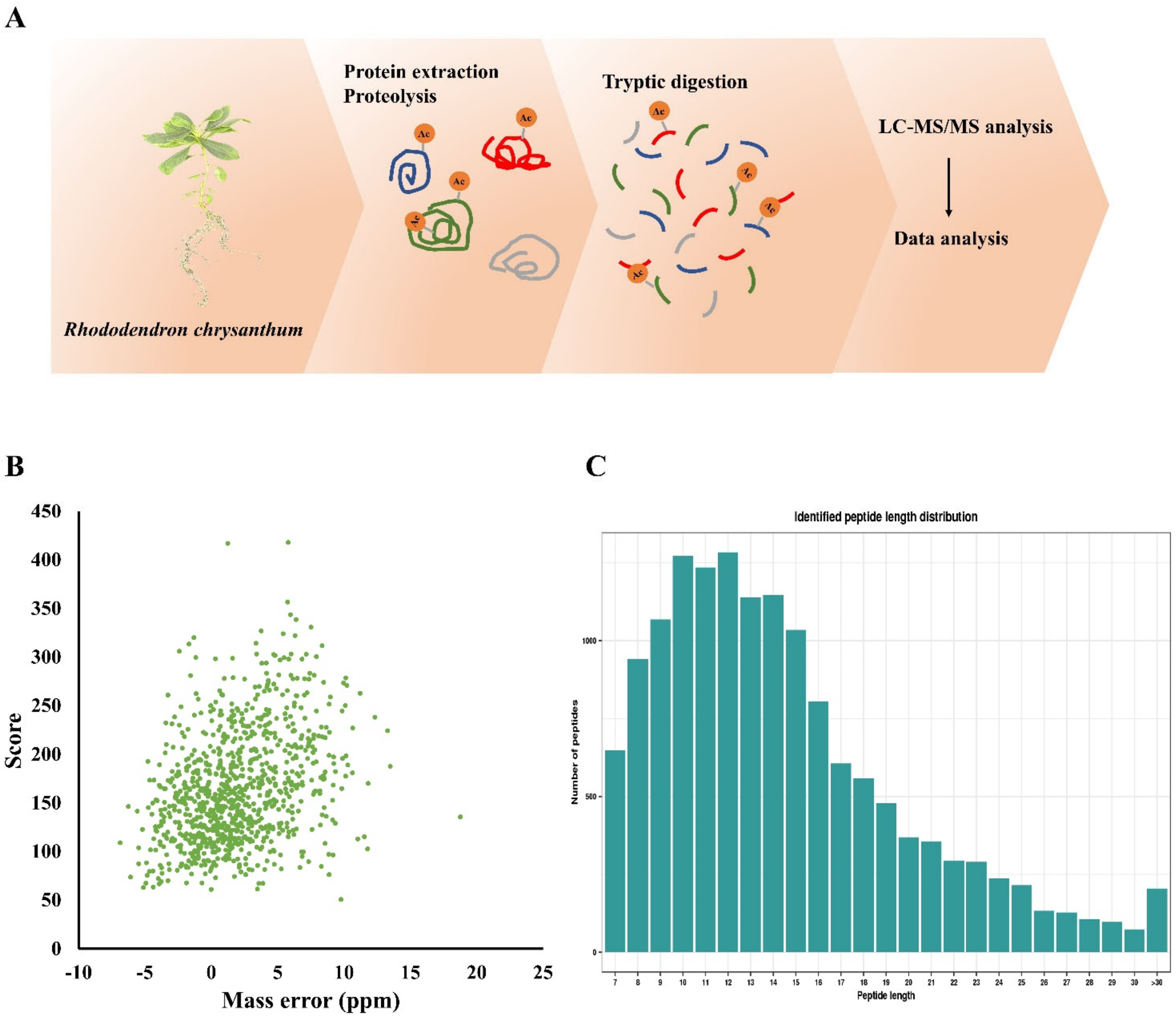

Figure S1. Identification of lysine acetylation proteome in *Rhododendron chrysanthum*. (A) 4D-label free experimental flowchart. (B) Mass error distribution of whole identified polypeptides. (C) The length distribution of identified peptide.

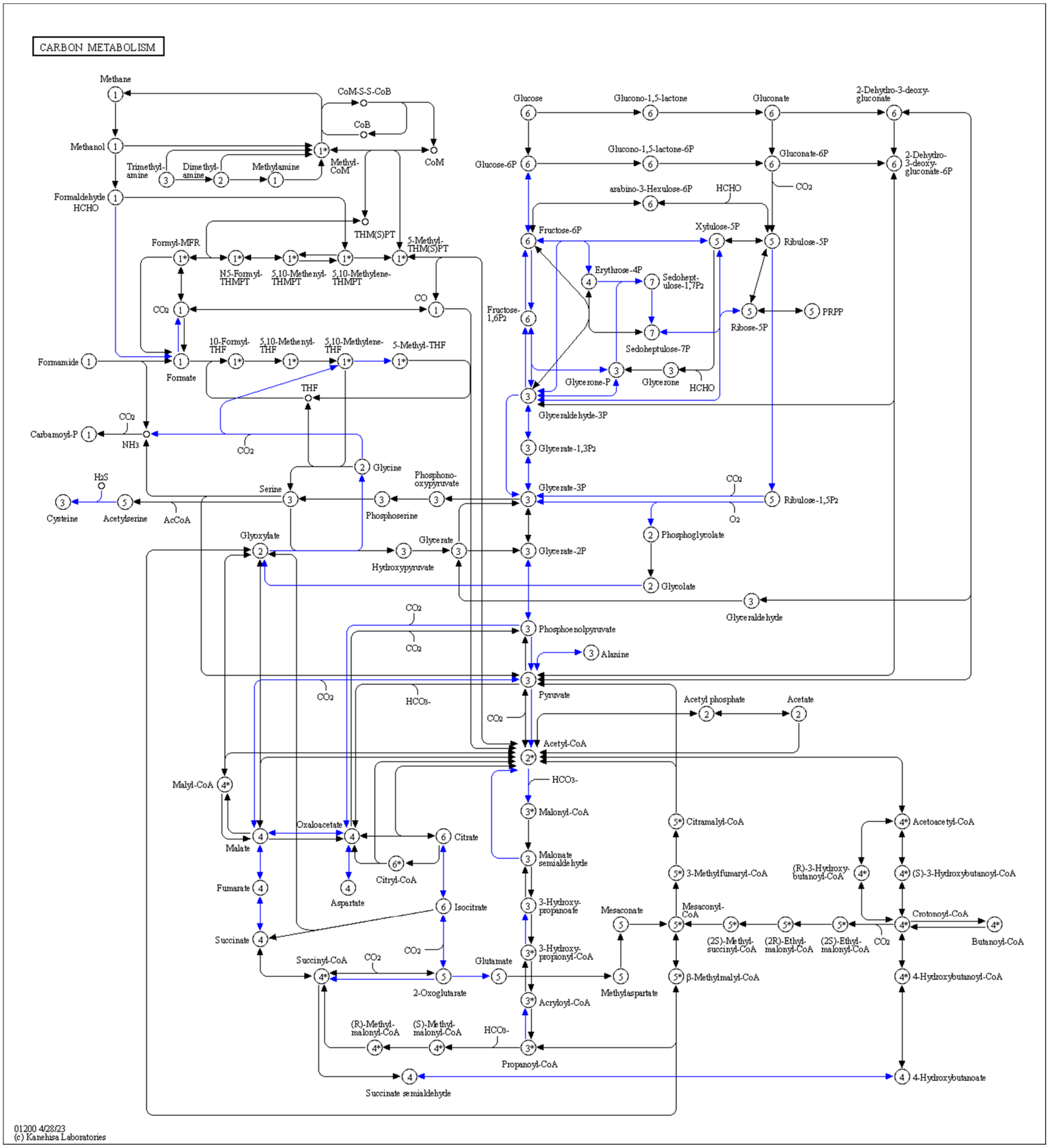

Figure S2. KEGG pathway enrichment analysis of the acetylated proteins in carbon metabolism. The acetylated proteins are in blue.

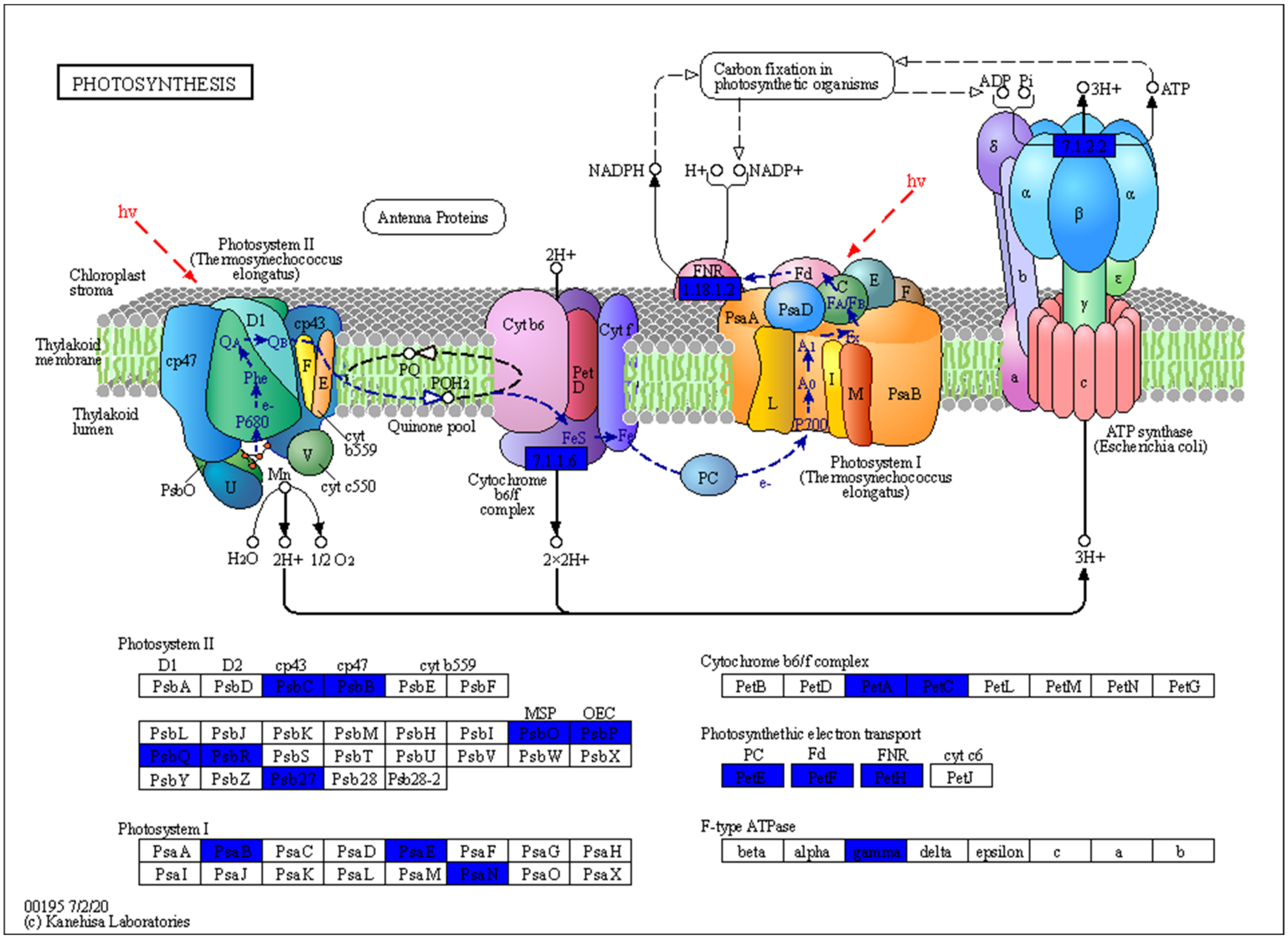

Figure S3. KEGG pathway enrichment analysis of the acetylated proteins in photosynthesis. The acetylated proteins are in blue.

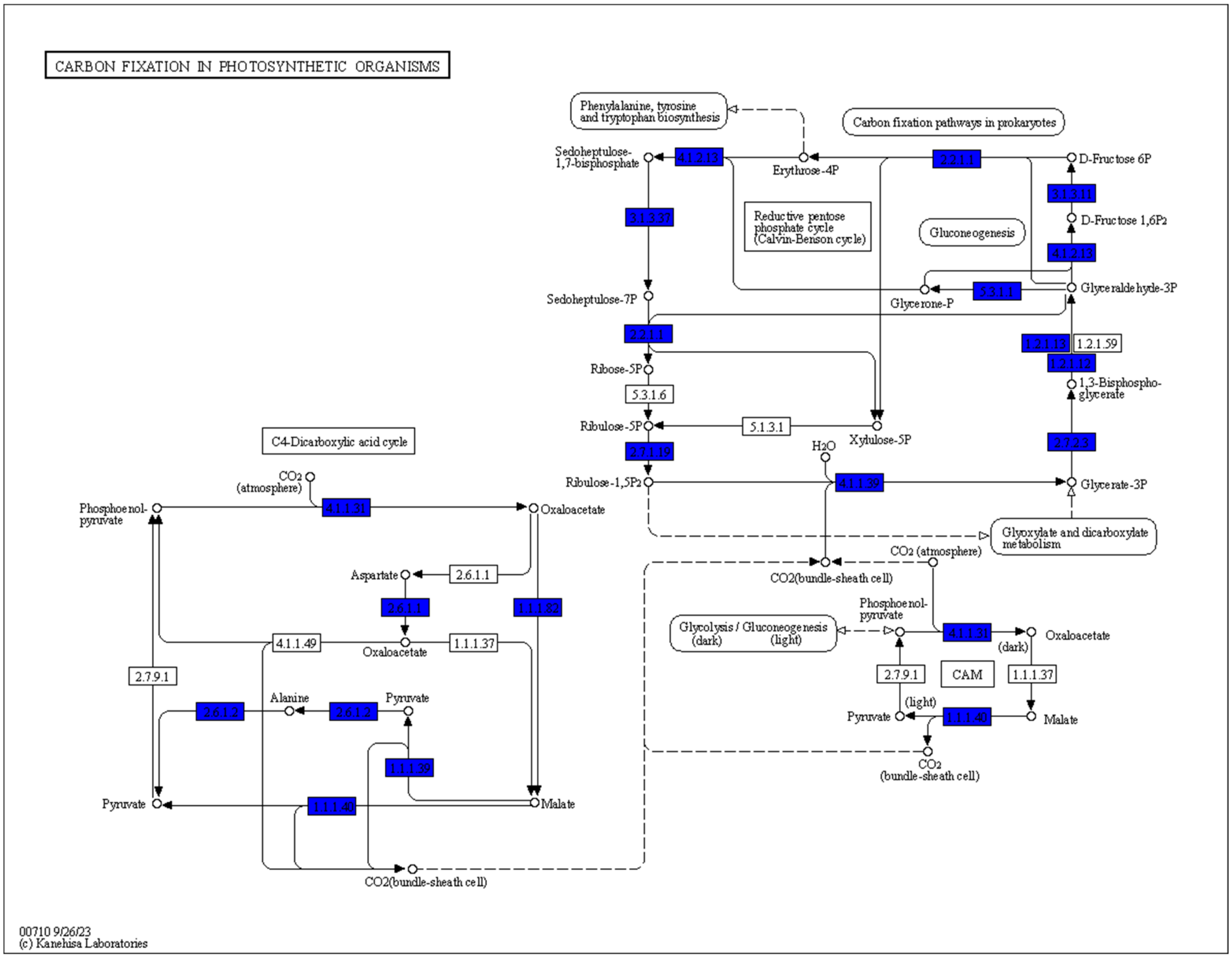

Figure S4. KEGG pathway enrichment analysis of the acetylated proteins in carbon fixation in photosynthetic organisms. The acetylated proteins are in blue.
